# Cortical auditory evoked potential with different speech stimuli in children with asymptomatic congenital cytomegalovirus infection

**DOI:** 10.1590/2317-1782/e20250108en

**Published:** 2026-04-10

**Authors:** Luara Rezende Madeira, Pamela Papile Lunardelo, Adriana Ribeiro Tavares

**Affiliations:** 1 Departamento de Ciências da Saúde, Faculdade de Medicina de Ribeirão Preto – FMRP, Universidade de São Paulo – USP - Ribeirão Preto (SP), Brasil.; 2 Departamento de Formação Específica em Fonoaudiologia, Instituto de Saúde de Nova Friburgo, Universidade Federal Fluminense – UFF - Nova Friburgo (RJ), Brasil.

**Keywords:** Congenital Cytomegalovirus Infection, Evoked Potentials, Auditory Processing, Child Development, Audiology

## Abstract

**Purpose:**

To characterize cortical auditory evoked potentials with speech stimuli in children with asymptomatic CMV infection compared to non-infected children with typical development of hearing, speech, and language.

**Methods:**

The sample included 23 children with asymptomatic congenital cytomegalovirus infection and 16 control children, matched by age, sex, head circumference, and socioeconomic status. Cortical Auditory Evoked Potentials were recorded using speech stimuli /da/ and /ga/ at 70 dB SPL, with electrodes placed at the vertex (Cz), forehead (Fpz), and earlobes (A1 and A2). The presence of components was analyzed and compared between groups using descriptive and inferential statistics.

**Results:**

The analysis of the waves indicated that the control group showed neural responses closer to those observed in mature auditory pathways, with the presence of N1 and P2 in the formation process, evidenced by clearer wave morphology. Regarding latency, significantly higher values were observed in the control group for the /ga/ syllable. The CMV group exhibited higher amplitudes for the N2 component, indicating greater neural effort in auditory stimulus discrimination.

**Conclusion:**

Children with asymptomatic cytomegalovirus may have alterations in central auditory processing with differences in detection and discrimination of acoustic cues compared to the control group. Cortical auditory evoked potentials are a tool to assess these alterations, and further research is needed to understand the effects of CMV infection on central auditory development.

## INTRODUCTION

Congenital cytomegalovirus (cCMV) is the most frequent intrauterine viral infection in childhood and has a significant impact on child development^(1,2)^. Vertical transmission of CMV may occur through contaminated amniotic fluid in contact with the fetus during pregnancy and has a reported worldwide prevalence of 0.2% to 1% or higher. cCMV infections may result in significant short-term morbidity and mortality in children who are symptomatic at birth and may result in long-term morbidity in asymptomatic children^(3)^. Although approximately 90% of children show no signs of congenital infection at the initial postnatal assessment (asymptomatic), 5%–15% develop late-onset abnormalities that compromise neurological development^(4)^, with sensorineural hearing loss (SNHL) being the most common sequela.

Studies investigating long-term impairments in asymptomatic children with cCMV have yielded contradictory findings and have primarily focused on cognitive functioning. Although some authors have found no differences in intelligence quotient (IQ) or other cognitive domains comparing children with asymptomatic cCMV infection and their non-infected peers^(5,6)^, other studies have suggested impairments attributable to asymptomatic cCMV infection^(7,8)^.

Although the integrity of the peripheral auditory pathway and hearing thresholds within normal limits are frequently observed in asymptomatic populations, evidence suggests that these children may present with alterations in central neural function that are not detectable through basic audiological assessment^(8–10)^. This hypothesis is based on the well-established affinity of cytomegalovirus for cells of the central nervous system (CNS), with the brain as the primary target organ^(9)^. CNS infections by neurotropic viruses tend to persist; therefore, intermittent reactivation of latent infections in the brain may occur long after birth in individuals with congenital CMV infection^(10)^.

Cortical auditory evoked potentials (CAEPs) arise from the neuroelectric activity of the primary and secondary auditory cortices and can provide information about biological processes involved in auditory processing and neural integrity of the central auditory nervous system^(11)^. CAEPs offer a more detailed view of the cortical neural processes underlying sound discrimination and integration, supporting the identification of specific aspects of speech signal encoding and guide planning and management of auditory rehabilitation^(12)^.

The ability to effectively process speech at the cortical level is directly associated with receptive and expressive language development^(13,14)^. Accordingly, variations in CAEP amplitude in response to contrasting speech stimuli have been widely used to predict developmental outcomes and evaluate the effects of interventions in infants and children with typical development^(15–17)^. Furthermore, this measure has been applied to populations with neurodevelopmental disorders associated with language and communication difficulties^(18–21)^.

The presence of P1–N1–P2 components with normal latencies and amplitudes reflects the efficient neural processing of acoustic signals at the auditory cortex level^(22)^. Predictive associations have been identified between specific infant CAEP peaks and language and cognitive skills in children at 3 and 4 years of age^(16)^.

Cortical potential is an essential tool for expanding the knowledge and understanding of the central auditory system and its capacity to process auditory information. This study aimed to characterize CAEPs elicited by contrasting speech sounds in children with asymptomatic cCMV compared with non-infected children with typical hearing, speech, and language development.

## METHODS

This analytical, observational, cross-sectional cohort study was conducted at a single assessment time point. The study was approved by the Institutional Review Board under protocol number 4.445.527. Written informed consent was obtained from the legal guardians of all participants.

### Participants

The sample comprised 23 children with asymptomatic cCMV infection (mean age: 7.87 ± 1.15 SD years; 10 girls and 13 boys), with a mean head circumference of 53.26 ± 1.82 SD cm and a mean socioeconomic classification score of 24.56 ± 6.33 SD. This group was compared with 16 children who tested negative for cCMV and presented typical hearing, speech, and language development (mean age: 7.88 ± 0.94 SD years; 8 girls and 8 boys), with a mean head circumference of 52.30 ± 1.57 SD cm and a mean socioeconomic classification score of 22.93 ± 5.32 SD. Groups were matched for sex, age, head circumference, and socioeconomic status.

Children from both groups (cCMV and control) were part of a previous study conducted by a research team (Brazilian Study on Congenital Cytomegalovirus, Hearing, and Secondary Maternal Infection–BraCHS). The presence or absence of congenital CMV infection was determined by salivary CMV DNA screening using the polymerase chain reaction (PCR). Positive salivary test results were confirmed using urine samples collected during the first week after birth.

Inclusion criteria for both groups were: hearing thresholds ≤ 15 dB HL (250–8000 Hz; Astera II audiometer, Madsen), type A tympanometric curve^(23)^ with the presence of ipsilateral and contralateral acoustic reflexes (500, 1000, 2000, and 4000 Hz; Otoflex 100 middle ear analyzer, Madsen), and absence of central nervous system infection, congenital malformations, genetic syndromes, or microcephaly.

### Procedures

Data were collected during a single assessment session lasting approximately 2 hours, including caregiver interviews and evaluation of peripheral and central auditory functions. Past and current information regarding the child’s neurodevelopment was obtained in addition to the administration of the Brazilian Economic Classification Criterion Questionnaire (ABEP)^(24)^.

### Cortical Auditory Evoked Potentials - CAEP

CAEPs were recorded using the SmartEP system (Intelligent Hearing Systems®, two-channel), calibrated in hearing level (dB HL). The participants were seated in a reclining chair inside a sound-treated booth, watched silent videos, and were instructed to remain awake throughout the examination. Skin preparation with 70% alcohol and abrasive gel (NuPrep®) preceded electrode placement. The electrodes were positioned as follows: active electrode at the vertex (Cz), ground electrode at the lower forehead (Fpz), and reference electrodes on the right (A2) and left (A1) earlobes.

The speech stimuli /da/ and /ga/, generated by the equipment (Table 1), were presented binaurally at 70 dB HL via inserted earphones (ER-3A), with equal probability (50% for each syllable) and in a randomized order. The stimulus presentation rate was 1.1/s, with a band-pass filter of 0.1–30 Hz and alternating polarity. EEG responses were amplified with a gain of 50,000 using a 500 ms analysis window and 140 sweeps (70 per syllable).

**Table 1:** Characteristics of the speech stimuli used

Parameter	/da/	/ga/
Stimulus duration (ms)	206,275	213,250
Consonant duration (ms)	9	38
Vowel duration (ms)	174	153
ISI	702,725	695,750
Pitch (onset–offset in Hz)	109,1–102,1	99,4–100,0
Formants (Hz)		
F1	732	775
F2	1335	1421
F3	2498	2242
F4	3058	3187
F5	3828	4613

**Caption:** ms = milliseconds; ISI = interstimulus interval; Hz = Hertz (cycles per second). Source: IHS Speech Stimuli

The components P1, N1, P2, and N2 were analyzed for all recordings within the 60–300 ms time window^(25)^, considering latency (ms) and amplitude (microvolts, µV). The recordings were analyzed individually and blindly by two evaluators with expertise in electrophysiology for component identification and marking. In cases of disagreement, a third evaluator made a final decision. The P1 component was defined as the first robust positive cortical wave at approximately 60 ms, N1 as the subsequent negative trough following P1, P2 as the most robust positive wave following N1, and N2 as the negative trough following P2.

### Statistical analysis

Statistical analyses were performed using the Statistical Package for the Social Sciences (SPSS), v.20.0. The results are expressed as descriptive measures of the mean and standard deviation. The Shapiro–Wilk test was used to assess data normality and determine the most appropriate statistical tests for the sample.

A linear mixed-effects model was used to compare the effects of different factors on the latency and amplitude parameters. The model included fixed factors of group (cCMV vs. control), syllable (/da/ and /ga/), and component (P1, N1, P2, and N2) as well as random effects for latency and amplitude to investigate whether neural responses to stimuli differed according to the group or syllable conditions.

## RESULTS

**Figure 1:**
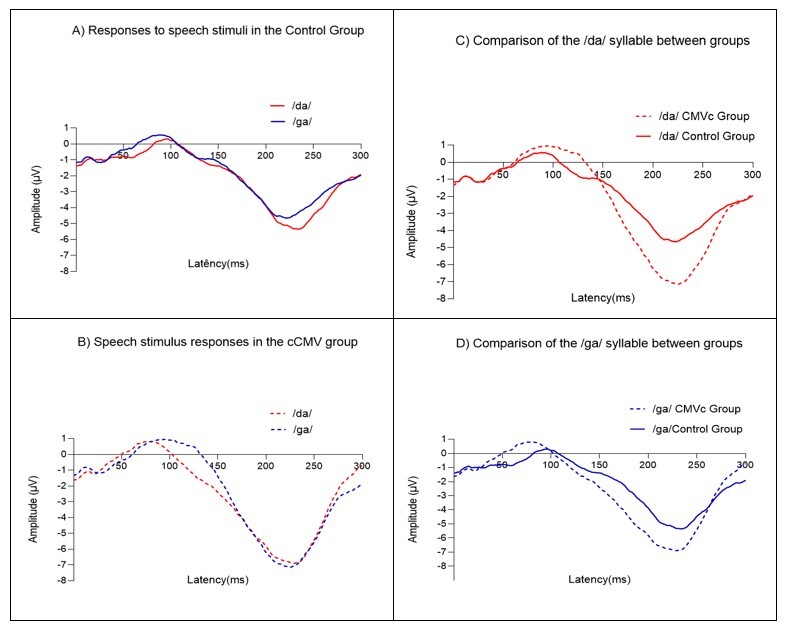
Grand average of the cortical auditory evoked potential for the different groups and speech stimuli

Figure 1 illustrates the average cortical auditory evoked potential (CAEP) waveforms elicited by the /da/ and /ga/ syllables for both groups and a comparison between them. Analysis of panels A and B indicated that the control group exhibited neural responses closer to those observed in mature auditory pathways, with the presence of N1 and P2 components during the process of maturation, as evidenced by a clearer waveform morphology. In contrast, in the cCMV group, neural responses were mainly limited to the P1 and N2 components. In the between-group comparison of contrasting syllables (panels C and D), the N2 component showed a greater amplitude for both syllables (/da/ and /ga/) in the cCMV group.

**Caption:** ms = milliseconds; MLM = Linear Mixed Model, between-group comparison

The descriptive values of latency and amplitude for the P1, N1, P2, and N2 components of the /da/ and /ga/ syllables, as well as the between-group comparisons, are presented in Tables 2 and 3.

**Table 2:** Description and comparison of latencies (P1, N1, P2, and N2) for the /da/ and /ga/ syllables between children with asymptomatic cCMV (n = 23) and the control group (n = 16)

	Control Group		cCMV Group		MLM Post-hoc (Sidak)
	Latency (ms)		Latency (ms)		
	Mean	Standard deviation	Mean	Standard deviation	p-value
P1					
/da/	101,77	32,75	97,55	25,00	0,58
/ga/	108,18	29,59	84,79	17,83	**0,005** *
N1					
/da/	139,90	31,70	135,57	27,25	0,63
/ga/	141,83	19,93	131,13	18,78	0,21
P2					
/da/	160,67	30,43	158,00	18,92	0,86
/ga/	192,00	12,72	164,5	17,67	0,21
N2					
/da/	226,19	20,06	225,74	15,02	0,95
/ga/	234,00	13,82	220,00	16,02	0,04*

* Statistically significant

**Caption:** ms = milliseconds; MLM = Linear Mixed Model, between-group comparison

**Table 3:** Description and comparison of absolute amplitudes (P1, N1, P2, and N2) for the /da/ and /ga/ syllables between children with asymptomatic cCMV (n = 23) and the control group (n = 16)

	Control Group		cCMV Group		MLM Post-hoc (Sidak)
	Amplitude (μV)		Amplitude (μV)		
	Mean	Standard deviation	Mean	Standard deviation	p-value
P1					
/da/	1,78	0,65	2,48	1,30	0,27
/ga/	1,83	0,90	2,25	1,39	0,56
N1					
/da/	2,53	1,89	3,47	2,34	0,21
/ga/	2,42	1,12	3,11	2,95	0,33
P2					
/da/	0,86	0,75	0,77	0,79	0,95
/ga/	0,22	0,04	0,89	0,35	0,71
N2					
/da/	5,65	2,21	7,82	2,39	**0,00** ^*^
/ga/	5,76	5,85	7,37	2,23	0,007*

* Statistically significant

### Latency

Analysis using the linear mixed-effects model revealed a significant effect when comparing the mean latencies for /da/ and /ga/ syllables between groups (F(1,190) = 6.80, p = 0.010). Post hoc analysis indicated that for the P1 (F(1,190) = 7.89, p < 0.001) and N2 (F(1,190) = 4.01, p = 0.040) components, the mean latency was significantly longer in the control group for the /ga/ syllable for both components. No significant differences were observed in the latencies of the P1, N1, P2, or N2 components within groups.

### Amplitude

The cCMV group exhibited higher mean amplitudes across all neural components, and the statistical model revealed a significant effect on the comparison of mean amplitudes between the groups (F(1,190) = 0.89, p = 0.018). Post hoc analysis revealed that the mean amplitude was significantly greater in the cCMV group for the N2 component for both /da/ (F(1,190) = 13.57, p < 0.001) and /ga/ syllables (F(1,190) = 7.45, p = 0.007). No significant differences were observed in the amplitudes of the P1, N1, and P2 components within the groups.

## DISCUSSION

CAEPs are widely used to assess central auditory processing and cortical skills associated with attention, auditory recognition, and discrimination^(25)^. These functions are fundamental to auditory information processing and are highly relevant in the investigation of pediatric populations in typical and atypical auditory developmental contexts^(25,26)^. Maturation of the central auditory system is characterized by developmental changes that begin in early childhood and stabilize in adulthood in a gradual and linear manner^(12,18,27)^.

The P1–N1–P2 components are referred to as exogenous or sensory potentials because they reflect the acoustic and temporal characteristics of the stimulus^(28)^. In contrast, the N2 is considered a mixed potential, presenting characteristics related to both exogenous and endogenous responses involved in the reception and interpretation of the physical and acoustic properties of the auditory stimulus. This component is influenced by discrimination task demands and attentional state, representing activity in the supratemporal auditory cortex^(29,30)^. The presence of P1–N1–P2 components with normal latencies and amplitudes reflects efficient neural processing of acoustic signals at the auditory cortex level^(22)^. This waveform complex provides information regarding the arrival of the auditory stimulus in the cortex and onset of cortical processing^(31)^.

The P1 component is considered a biomarker of auditory detection ability, and its analysis allows inferences regarding the maturational status of the central auditory pathways^(32)^. This component measures changes in latency and amplitude according to the acoustic characteristics of the stimulus, reflecting sensory encoding and is part of the ascending auditory pathway^(33)^.

The N1 component is a marker of the attentional auditory cortical activity related to the perception of sound onset. The stimulus type (tone or speech), intensity, duration, and presentation rate may influence the presence, latency, and amplitude of the N1 component^(34)^. Although N1 is age-dependent and exhibits an inconsistent pattern between the ages of 3 and 8 years^(35)^, it is considered a reliable indicator for investigating the neural timing of speech processing^(36)^.

Studies have shown that increases in P2 amplitude coincide with improved perceptual performance; however, little is known about the functional significance and neural generators of the P2 auditory response or whether it may serve as a biological marker of auditory learning^(37)^. The N1–P2 complex can be observed from approximately 6 years of age and should be well defined in adulthood^(38)^.

Analysis of the morphology of the P1–N1–P2 complex (Figures 11B) showed that the control group presented a more clearly defined N1–P2 complex for both speech stimuli. Notably, the groups were age-matched; nevertheless, despite similar ages, the cCMV group exhibited an ill-defined N1–P2 complex, suggesting a maturational delay in the auditory pathway^(39)^.

The presence of this ill-defined N1–P2 complex suggests an increased synchronization of neuronal activity and the establishment of effective structural networks for the initial detection and discrimination of auditory stimuli^(40)^. Therefore, the absence of age-expected components or poor waveform morphology may serve as warning signs of potential auditory perceptual difficulties in children with cCMV infection.

The P1–N1–P2 complex is sensitive to the acoustic parameters of speech signals and is frequently used to investigate the neural detection of spectral and temporal cues in populations with speech and language difficulties^(41)^.

The /ga/ syllable presented longer total duration (213.3 ms), longer consonant duration (38 ms), and shorter interstimulus interval (ISI; 695.8 ms) compared with the /da/ syllable, which had values of 206.3 ms, 9 ms, and 702.7 ms, respectively.

In the control group, the mean P1 and N2 latencies for /da/ were shorter than those for /ga/, which was consistent with the acoustic characteristics (duration) of the presented stimuli. Compared to the control group, children with cCMV showed shorter mean P1 and N2 latencies for both syllables. Although the control group demonstrated the ability to detect and discriminate between different acoustic cues, the cCMV group seemed less effective at detecting syllable duration patterns, which may suggest altered temporal perception. Alterations in the timing of speech sound differentiation have been previously reported in children with language and communication difficulties associated with other neurodevelopmental conditions^(19,42)^.

The N2 component was strongly influenced by stimulus intensity, probability of occurrence, task difficulty in determining the differences between stimuli, and the participant’s attentional state. N2 may be related to inhibitory responses and may serve as an important marker of auditory processing disorders in children^(43)^. Additionally, this component is commonly associated with the pre-attentional processes involved in the conscious discrimination of auditory stimuli.

The cCMV group exhibited greater N2 amplitudes for both presented stimuli (/da/ and /ga/) than the control group. Amplitude measures reflect the magnitude of the neural activity elicited by a sound stimulus and the number of nerve fibers recruited during auditory processing along the auditory pathway^(29)^.

With the maturation of auditory pathways, a reduction in waveform amplitude occurs because of increased neural efficiency^(44)^. Thus, the greater N2 amplitude observed in children with cCMV may be associated with auditory pathway immaturity, requiring greater neural effort and broader neural activation to discriminate the stimulus, thereby increasing the waveform amplitude.

CAEPs reflect the activity of excitatory postsynaptic potentials in the thalamus and superior auditory cortex (primary auditory cortex and association areas)^(45)^. Maturation of the thalamocortical portions of the central auditory system can be assessed by recording age-related changes in the neurophysiological responses induced by auditory stimulation^(32)^.

Congenital CMV infection may cause thalamic alterations that are identifiable on fetal ultrasonography. Previous studies have suggested that the thalamus may be the brain region initially affected by CMV infection, highlighting its vulnerability to CMV infection during early development^(46)^.

Therefore, children with cCMV may be more vulnerable to neurodevelopmental alterations and, consequently, are at a risk of central auditory processing disorders, as speech and language development depend on the perception and discrimination of a wide spectrum of speech sounds.

Children with asymptomatic cCMV may present with hearing loss at birth or late onset^(47)^. However, literature regarding central auditory system alterations and the impact of cCMV on auditory processing remains scarce.

No latency alterations in auditory brainstem responses have been observed during the first year of life in children with cCMV^(48)^; however, there is limited information from studies applying CAEPs in this population. This underscores the need for further investigations to deepen our understanding of the effects of cCMV on central auditory processing. Future research should explore the impact of cCMV on the maturation and functioning of auditory pathways at the cortical level, thereby contributing to a more comprehensive understanding and development of more effective diagnostic and therapeutic strategies.

## CONCLUSION

Children with asymptomatic cCMV may be at risk for alterations in central auditory processing. Our findings demonstrated differences in auditory cortical responses in these children, characterized by ill-defined waveform morphology, shorter N1 and P2 latencies, and greater N2 amplitude, when compared with age-matched children without viral exposure. This study reinforces the importance of CAEPs as a tool for assessing these alterations and highlights the need for future research to better understand the effects of cCMV on central auditory development.
