# Supplementary material for: Cortical auditory evoked potential with different speech stimuli in children with asymptomatic congenital cytomegalovirus infection
Source: Codas. 2026 Apr 10;38(2):e20250108. doi: 10.1590/2317-1782/e20250108en (PMC13075846; doi:10.1590/2317-1782/e20250108en)
Supplement: Translation

# Translation: Potencial evocado auditivo cortical com diferentes estímulos de fala em crianças com citomegalovirose congênita assintomática

**DOI:** 10.1590/2317-1782/e20250108pt

**Authors:** Luara Rezende Madeira, Pamela Papile Lunardelo, Adriana Ribeiro Tavares

**Affiliations:** 1 Departamento de Ciências da Saúde, Faculdade de Medicina de Ribeirão Preto – FMRP, Universidade de São Paulo – USP - Ribeirão Preto (SP), Brasil.; 2 Departamento de Formação Específica em Fonoaudiologia, Instituto de Saúde de Nova Friburgo, Universidade Federal Fluminense – UFF - Nova Friburgo (RJ), Brasil.

**Keywords:** Infecção por Citomegalovírus Congênita, Potenciais Evocados, Processamento Auditivo, Desenvolvimento Infantil, Audiologia

## Abstract

**Objetivo:**

Caracterizar os potenciais evocados auditivos corticais (PEAC) com estímulos de fala em crianças com CMVc assintomático comparando-as a crianças não infectadas com desenvolvimento típico de audição, fala e linguagem.

**Método:**

A amostra incluiu 23 crianças com infecção assintomática por citomegalovírus congênito e 16 crianças controle, pareadas por idade, sexo, perímetro cefálico e classificação socioeconômica.O Potencial Evocado Auditivo Cortical foi registrado com estímulos de fala /da/ e /ga/ a 70 dBNA, utilizando eletrodos posicionados no vértice (Cz), na região frontal (Fpz) e lóbulos (A1 e A2). A presença dos componentes foi analisada e comparada entre os grupos por meio de estatística descritiva e inferencial.

**Resultados:**

A análise das ondas indicou que o grupo controle apresentou respostas neurais mais próximas daquelas observadas em vias auditivas maduras, com a presença dos componentes N1 e P2 em processo de formação, evidenciada pela morfologia mais nítida nas ondas.Em relação à latência, observaram-se valores significativamente maiores no grupo controle para a sílaba /ga/.O grupo CMVc exibiu amplitudes superiores para o componente N2, indicando maior esforço neural na discriminação do estímulo auditivo.

**Conclusão:**

Crianças com citomegalovírus assintomático podem apresentar alterações no processamento auditivo central, com diferenças na detecção e discriminação e pistas acústicas em relação ao grupo controle. O Potencial Evocado Auditivo Cortical é uma ferramenta para avaliar essas alterações, sendo necessárias mais pesquisas para compreender os efeitos do CMVc no desenvolvimento auditivo central.

## INTRODUÇÃO

A infecção por citomegalovírus congênito (CMVc) é a mais frequente das infecções virais intrauterinas na infância, implicando em alterações significativas para o desenvolvimento infantil^(1,2)^. A transmissão vertical do CMV pode ocorrer por líquido amniótico contaminado em contato com o feto durante a gravidez, com prevalência que varia de 0,2% a 1% ou mais no âmbito mundial O CMVc pode resultar em morbidade e mortalidade significativa em curto prazo em crianças sintomáticas ao nascimento e, morbidade em longo prazo em crianças assintomáticas^(3)^. Embora, 90% das crianças não apresentem sinais de infecção congênita na primeira avaliação logo após o nascimento (assintomática), 5% a 15% delas desenvolvem anormalidades tardias que comprometem seu desenvolvimento neurológico^(4)^ sendo a perda auditiva sensorioneural (PASN), a sequela mais comum.

Estudos sobre o comprometimento de longo prazo em crianças assintomáticas com CMVc são contraditórios e focados na análise do funcionamento cognitivo.

Mesmo que alguns autores não tenham encontrado diferenças no quociente de inteligência (QI) ou em vários domínios cognitivos entre crianças com CMVc assintomático em comparação com crianças não infectadas^(5,6)^, outros estudos sugerem comprometimentos atribuíveis em crianças assintomáticas com CMVc^(7,8)^.

Embora a integridade da via auditiva periférica e a presença de limiares auditivos dentro, dos padrões de normalidade sejam características frequentemente observadas na população assintomática, evidências sugerem que essas crianças podem apresentar alterações na função neural central não identificáveis na avaliação auditiva básica^(8–10)^. A hipótese baseia-se no fato de que o citomegalovírus tem afinidade bem conhecida pelas células do Sistema Nervoso Central (SNC), sendo o cérebro um órgão-alvo^(9)^. A infecção do SNC por vírus neurotrópico tende a se tornar uma neuroinfecção persistente. Portanto, a reativação intermitente da infecção latente no cérebro pode ocorrer muito tempo após o nascimento em indivíduos com infecção congênita por CMV^(10)^.

Os potenciais evocados auditivos corticais (PEAC) resultam da atividade neuroelétrica do córtex auditivo primário e secundário e podem fornecer informações sobre os processos biológicos envolvidos no processamento e na integridade neural do sistema nervoso auditivo central^(11)^. Eles fornecem uma visão mais detalhada dos processos neurais corticais subjacentes à discriminação e integração sonora, auxiliando a identificação de aspectos específicos da qualidade da codificação do sinal de fala e orientação no planejamento e gerenciamento da reabilitação auditiva^(12)^.

A capacidade de processar efetivamente a fala em nível cortical está diretamente associada ao desenvolvimento da linguagem receptiva e expressiva^(13,14)^. Assim, a variação na amplitude do PEAC em resposta a estímulos de fala contrastantes tem sido amplamente utilizada para prever desfechos no desenvolvimento e para avaliar os efeitos de intervenções em bebês e crianças com desenvolvimento típico^(15–17)^. Além disso, essa medida tem sido aplicada em populações com diversos transtornos do neurodesenvolvimento associados a dificuldades de linguagem e comunicação^(18–21)^.

A presença dos componentes P1-N1-P2 com latências e amplitudes normais,reflete a eficiência do processamento neural do sinal acústico no nível do córtex auditivo^(22)^. Associações preditivas foram encontradas entre picos específicos do PEAC infantil e habilidades de linguagem e cognitivas aos 3 e 4 anos de idade^(16)^.

Os potenciais corticais oferecem uma ferramenta essencial para ampliar o conhecimento e a compreensão sobre sistema auditivo central e sua capacidade de processar informações auditivas. O objetivo deste estudo foi caracterizar os PEACs para sons de fala contrastantes nas crianças com CMVc assintomático, em comparação a crianças não infectadas com desenvolvimento típico de audição, fala e linguagem.

## MÉTODO

Trata-se de um estudo analítico, observacional, de coorte transversal, realizado em um único momento de avaliação, aprovado pelo Comitê de Ética, sob o parecer de número 4.445.527. O termo de consentimento livre e esclarecido (TCLE) foi obtido para todos os participantes.

### Participantes

A amostra foi composta por 23 crianças com infecção assintomática por citomegalovírus congênito (CMVc) (idade média: 7,87 ± 1,15 dP anos; 10 meninas e 13 meninos), com perímetro cefálico médio de 53,26 ± 1,82 dP cm e pontuação média na classificação socioeconômica de 24,56 ± 6,33 dP. Esse grupo foi comparado a 16 crianças com resultado negativo para CMVc e desenvolvimento típico de audição, fala e linguagem (idade média: 7,88 ± 0,94dP anos; 8 meninas e 8 meninos), apresentando perímetro cefálico médio de 52,3 ± 1,57 dP cm e pontuação média na classificação socioeconômica de 22,93 ± 5,32 dP. Os grupos foram pareados por sexo, idade, perímetro cefálico e classificação socioeconômica.

As crianças de ambos os grupos (CMVc e controle) fazem parte de um estudo anterior dos pesquisadores (Estudo Brasileiro sobre Citomegalovirose congênita, Audição e Infecção Secundária Materna-BraCHS). A presença ou ausência do CMV congênito foi estabelecida pela triagem do DNA-CMV na saliva, utilizando a reação em cadeia da polimerase (PCR). Os resultados positivos na saliva foram confirmados por testes de urina coletados nas primeiras semanas após o nascimento.

Os critérios de inclusão para ambos os grupos foram: limiares auditivos até 15dBNA (250 a 8000 Hz- Audiômetro Astera II-Madsen), curva tipo A^(23)^ com presença dos reflexos acústicos ipsi e contralaterais (frequências de 500, 1000, 2000 e 4000 Hz- Analisador de Orelha Média Otoflex 100- Madsen), ausência de infecção do sistema nervoso central, malformação congênita, síndrome genética e microcefalia.

### Procedimentos

Os dados foram coletados em um único momento de avaliação, com duração de aproximadamente 2 horas, incluindo entrevista com os cuidadores e avaliação da audição periférica e central. Foram coletadas informações pregressas e atuais da criança sobre o neurodesenvolvimento,além da aplicação do questionário para Critério de Classificação Econômica Brasil (ABEP)^(24)^.

### Potencial Evocado Auditivo Cortical - PEAC

Para o registro do PEAC foi utilizado o equipamento Smart EP, da Intelligent Hearing System®, com dois canais, calibrado em nível de audição (dBnNA). Os participantes foram posicionados em poltrona reclinável dentro de cabina acústica, assistindo a vídeos silenciosos e orientados a não dormir durante a realização do exame. A limpeza da pele com álcool 70% e gel abrasivo (NuPrep®) antecedeu a colocação dos eletrodos, que foram posicionados nas seguintes configurações: um eletrodo ativo colocado no vértice da cabeça (Cz); um eletrodo terra posicionado na região inferior da fronte (Fpz), e dois eletrodos referência posicionados na região dos lóbulos direito (A2) e esquerdo (A1).

Os estímulos de fala /da/ e /ga/, gerados pelo equipamento (Tabela 1), foram apresentados a 70dBNA, com fones de inserção (ER3A) no modo binaural, com igual probabilidade (50% para cada sílaba), de modo aleatório. A taxa de apresentação dos estímulos foi de 1,1/s, filtro de 0,1 a 30 Hz, na polaridade alternada. O EEG foi amplificado com ganho de 50.000 em janela de apresentação de 500 ms, com 140 promediações (70 para cada sílaba).

**Tabela 1:** Características dos estímulos de fala utilizados estímulos de fala utilizados

Parâmetros	/da/	/ga/
Duração do estímulo (ms)	206,275	213,250
Duração da consoante (ms)	9	38
Duração da vogal (ms)	174	153
ISI	702,725	695,750
Pitch (início-fim em Hz)	109,1 – 102,1	99,4 – 100,0
Formantes (Hz)		
F1	732	775
F2	1335	1421
F3	2498	2242
F4	3058	3187
F5	3828	4613

**Legenda:** ms = milissegundo; ISI = intervalo inter-estímulo; Hz = Hertz (ciclo por segundo). Fonte: *IHS Speech Stimuli*

Os componentes (P1, N1, P2 e N2) analisados para todos os registros obtidos, no intervalo entre 60 e 300 milissegundos (ms)^(25)^, quanto à sua latência (ms) e amplitude (microvolt - μV). Os registros foram analisados individualmente, de maneira cega, por dois avaliadores com experiência em eletrofisiologia, para identificação e marcação dos potenciais. Em caso de divergência, um terceiro avaliador decidiu pela marcação dos potenciais. O componente P1 foi considerado como a primeira onda cortical positiva mais robusta em torno de 60ms, o N1 foi analisado como o vale subsequente à onda P1, com maior negatividade, a resposta de P2 foi identificada como a onda positiva mais robusta após N1; e a N2, como o vale subsequente à onda P2, com maior negatividade.

### Análise estatística dos dados

A análise estatística foi realizada por meio do programa *Statistical Package for the Social Sciences* (SPSS) versão 20.0. Os resultados foram expressos através das medidas descritivas de média e desvio-padrão. Foi aplicado o teste de normalidade shapiro-wilk para a decisão do teste estatístico mais robusto para a amostra.

Neste estudo, foi utilizado um modelo linear misto para comparar os efeitos de diferentes fatores sobre os parâmetros de latência e amplitude. O modelo foi estruturado para considerar os fatores fixos: grupos (CMVc x Controle), sílabas (/da/ e /ga/) e componentes (P1- N1- P2- N2) e, os fatores aleatórios, como a latência e a amplitude, para investigar se a resposta neural ao estímulo difere de acordo com as condições dos grupos ou das sílabas.

## RESULTADOS

Na Figura 1, ilustramos a resposta média geral (*grand average*) das ondas do PEAC para as sílabas /da/ e /ga/ dos grupos, bem como a comparação entre eles. A análise dos gráficos A e B reflete que o grupo controle apresenta respostas neurais mais próximas daquelas observadas em vias auditivas maduras, com a presença de N1 e P2 em processo de formação, evidenciada pela morfologia mais nítida dos componentes. Em contraste, no grupo CMVc, as respostas neurais limitam-se aos componentes P1 e N2. Na comparação entre os grupos, para as sílabas contrastantes (gráficos C e D), é possível observar que o componente N2 apresenta maior amplitude para ambas as sílabas (/da/ e /ga/) no grupo CMVc.

**Figura 1:** *Grand average* do potencial evocado auditivo cortical para os diferentes grupos e estímulos de fala

**Legenda:** ms = milisegundos; MLM = Modelo Linear Misto comparação entre os grupos

A descrição dos valores de latência e amplitude dos componentes P1,N1,P2 e N2 para as sílabas /da/ e /ga/, assim como a comparação entre os grupos, está apresentada nas Tabelas 2 e 3.

**Tabela 2:** Descrição e comparação das latências (P1, N1, P2 e N2) para as sílabas /da/ e /ga/ entre as crianças com CMVc assintomático (n=23) e grupo controle (n= 16)

	Grupo Controle		Grupo CMVc		MLM Post-hoc (Sidak)
	Latência(ms)		Latência(ms)		
	Média	Desvio Padrão	Média	Desvio Padrão	p-valor
P1					
/da/	101,77	32,75	97,55	25,00	0,58
/ga/	108,18	29,59	84,79	17,83	**0,005** ^*^
N1					
/da/	139,90	31,70	135,57	27,25	0,63
/ga/	141,83	19,93	131,13	18,78	0,21
P2					
/da/	160,67	30,43	158,00	18,92	0,86
/ga/	192,00	12,72	164,5	17,67	0,21
N2					
/da/	226,19	20,06	225,74	15,02	0,95
/ga/	234,00	13,82	220,00	16,02	0,04*

* Valores estatisticamente significantes

**Legenda:** ms = milisegundos; MLM = Modelo Linear Misto comparação entre os grupos

**Tabela 3:** Descrição e comparação das amplitudes absolutas (P1, N1, P2 e N2) para as sílabas /da/ e /ga/ entre as crianças com CMVc assintomático(n=23) e grupo controle (n= 16)

	Grupo Controle		Grupo CMVc		MLM Post-hoc (Sidak)
	Amplitude (μV)		Amplitude (μV)		
	Média	Desvio Padrão	Média	Desvio Padrão	p-valor
P1					
/da/	1,78	0,65	2,48	1,30	0,27
/ga/	1,83	0,90	2,25	1,39	0,56
N1					
/da/	2,53	1,89	3,47	2,34	0,21
/ga/	2,42	1,12	3,11	2,95	0,33
P2					
/da/	0,86	0,75	0,77	0,79	0,95
/ga/	0,22	0,04	0,89	0,35	0,71
N2					
/da/	5,65	2,21	7,82	2,39	**0,00** ^*^
/ga/	5,76	5,85	7,37	2,23	0,007*

* Valores estatisticamente significantes

### Latência

A análise do modelo linear misto revelou um efeito significativo quando da comparação das médias de latência das sílabas /da/ e /ga/ entre os grupos (F(1,190)=6,80,p= 0,010). O teste post hoc indicou que, para os componentes P1 (F(1,190)=7,89,p= 0,00) e N2 (F(1,190)=4,01,p= 0,04), a média de latência foi significativamente maior no GC para a sílaba /ga/, em ambas as componentes. Não houve diferença significativa para as latências dos componentes P1, N1, P2 e N2 dentro dos grupos.

### Amplitude

O grupo CMVc apresentou maiores médias de amplitude para todas as componentes neurais, e o modelo estatístico revelou um efeito significativo na comparação das médias de amplitude entre os grupos (F(1,190)=0,89,p= 0,018). O teste post hoc indicou que a média de amplitude foi significativamente maior no grupo CMVc para o componente N2, tanto para a sílaba /da/ (F(1,190)=13,57p= 0,000) quanto para a sílaba /ga/ (F(1,190)=7,45,p= 0,007). Não houve diferença significativa para as amplitudes dos componentes P1, N1, P2 e N2 dentro dos grupos.

## DISCUSSÃO

O PEAC é uma ferramenta amplamente utilizada para avaliar o processamento auditivo central, bem como as habilidades corticais associadas à atenção, reconhecimento e discriminação auditiva^(25)^. Essas funções são fundamentais no processamento da informação auditiva e têm grande relevância na investigação de populações infantis, tanto em contextos de desenvolvimento auditivo típico quanto atípico^(25,26)^. A maturação do sistema auditivo central é marcada por modificações ao longo do desenvolvimento, iniciando-se já nos primeiros anos de vida e estabilizando-se na faixa etária adulta, ocorrendo de maneira gradativa e linear^(12,18,27)^.

Os componentes P1-N1-P2 são chamados exógenos ou sensoriais, por refletirem as características acústicas e temporais do estímulo^(28)^. Já o N2 é considerado um potencial misto, que possui características relativas às respostas exógena e endógena envolvidas na recepção e interpretação das informações físicas e acústicas do estímulo auditivo. Este componente sofre influências da tarefa de discriminação e de estado de atenção, representando a atividade do córtex auditivo supratemporal^(29,30)^. A presença dos componentes P1-N1-P2 em latências e amplitudes normais reflete a eficiência do processamento neural do sinal acústico no nível do córtex auditivo^(22)^. Esse complexo de ondas fornece informações sobre a chegada do estímulo auditivo ao córtex e o início do processamento cortical ^(31)^.

O componente P1 é considerado um biomarcador da habilidade de detecção auditiva, e sua análise permite inferir sobre o estado de amadurecimento das vias auditivas centrais^(32)^. Essa componente sofre alteração de latência e amplitude de acordo com as característica acústicas do estímulo, refletindo a codificação sensorial e parte da via auditiva ascendente^(33)^.

N1 é um marcador da atividade cortical auditiva atencional, relativo à percepção do início do som. O tipo do estímulo (tom ou fala), intensidade , duração e taxa de apresentação podem interferir na presença, latência ou amplitude do componente N1^(34)^. Embora N1 seja dependente da idade e apresente um padrão inconsistente entre os três e oito anos de idade^(35)^, esse componente é considerado um indicador confiável para investigar o tempo neural do processamento da fala^(36)^.

Estudos têm demonstrado que aumentos na amplitude da onda P2 coincidem com a percepção melhorada, entretanto, pouco se sabe sobre o significado funcional e os geradores neurais da resposta auditiva P2 e, se ela poderia ou não servir como um marcador biológico de aprendizagem auditiva^(37)^. Sabe-se que o complexo N1-P2 pode ser visualizado por volta dos seis anos de idade e deve estar bem definido na fase adulta^(38)^.

Analisando a morfologia do complexo P1-N1-P2 (Figuras 11B), identificamos no grupo controle, que o complexo N1-P2 apresentou morfologia mais nítida para ambos os estímulos de fala aplicados. É importante ressaltar que ambos os grupos foram pareados por idade e, mesmo com idade semelhante, o grupo com CMVc, apresentou indefinição do complexo N1-P2, sugerindo atraso maturacional da via auditiva^(39)^.

A presença do complexo sugere o aumento da sincronização da atividade neuronal e o estabelecimento de redes estruturais efetivas para a detecção e discriminação inicial do estímulo auditivo^(40)^. Assim, a ausência dos componentes já esperados para a idade ou uma morfologia ruim do complexo são alertas para a possibilidade de dificuldades perceptuais auditivas nas crianças com CMVc.

O complexo P1-N1-P2 tem se mostrado sensível aos parâmetros acústicos do sinal de fala e, frequentemente, é usado para o estudo da detecção neural de pistas espectrais e temporais em populações com dificuldades de fala^(41)^.

A sílaba /ga/ apresentou maior duração total (213,3 ms), maior tempo de duração da consoante (38ms) e menor tempo de ISI (695,8ms), em relação à sílaba /da/, com 206,3ms, 9ms e 702,7ms, respectivamente.

No grupo controle, foi possível observar que as médias de latência de P1 e N2 para /da/ foram menores que para /ga/, o que condiz com as características acústicas (tempo de duração) dos estímulos apresentados. Em comparação ao grupo controle, as crianças com CMVc apresentaram médias das latências de P1 e N2 menores para ambas as sílabas. Enquanto no grupo controle parece ter havido a habilidade de detecção e discriminação de diferentes pistas acústicas, o grupo com CMVc parece não detectar o padrão de duração das sílabas de forma tão efetiva*,* o que poderia sugerir uma percepção temporal alterada. Alterações no tempo de diferenciação dos sons da fala foram relatadas anteriormente em crianças com dificuldades de linguagem e comunicação devido a outras condições do neurodesenvolvimento^(19,42)^.

O componente N2 é fortemente influenciado pela intensidade e probabilidade de ocorrência do estímulo, pela dificuldade em determinar a diferença entre dois estímulos e pela atenção do sujeito. O componente N2 pode estar relacionado à resposta inibitória e pode ser um marcador importante de problemas em crianças com transtorno do processamento auditivo^(43)^.Além disso, esse componente é comumente associado a processos pré-atencionais envolvidos na discriminação consciente do estímulo sonoro.

O grupo CMVc apresentou amplitude maior para o componente N2 para ambos os estímulos apresentados (/da/ e /ga/) quando comparado ao grupo controle. As medidas de amplitude refletem a magnitude da atividade neural gerada frente a um estímulo sonoro e a quantidade de fibras nervosas recrutadas no processamento do estímulo ao longo da via auditiva^(29)^.

Com o amadurecimento das vias auditivas, ocorre uma redução na amplitude das ondas devido a uma maior eficiência neural^(44)^. É possível que a maior amplitude de N2 em crianças com CMVc esteja associada à imaturidade da via auditiva, demandando maior esforço e maior propagação da ativação neural para discriminar o estímulo, aumentando a amplitude da onda.

Os PEAC refletem a atividade de potenciais pós-sinápticos excitatórios no nível do tálamo e do córtex auditivo superior (córtex auditivo primário e áreas de associação^(45)^. A maturação das porções tálamo-corticais do sistema auditivo central pode ser avaliada pelo registro de mudanças relacionadas à idade nas respostas neurofisiológicas evocadas pela estimulação auditiva^(32)^.

A infecção congênita pelo CMV pode revelar alterações no tálamo identificadas pela ultrassonografia fetal. Estudo sugeriu que o tálamo pode ser a região cerebral inicialmente impactada pela infecção por CMV, enfatizando sua vulnerabilidade durante o desenvolvimento inicial^(46)^.

Assim, crianças com CMVc podem ser mais vulneráveis à alterações no neurodesenvolvimento e, consequentemente, estar em risco para alterações no processamento auditivo central, à medida que o desenvolvimento da fala e da linguagem depende da percepção e discriminação de um amplo espectro de sons de fala.

Crianças com CMVc assintomático podem apresentar perda auditiva ao nascimento ou de início tardio^(47)^. Entretanto, a literatura é escassa em relação às alterações do sistema auditivo central e o impacto causados pelo CMVc no processamento auditivo.

Não foram observadas alterações de latência nas respostas auditivas de tronco encefálico no primeiro ano de vida, em crianças com CMVc^(48)^, mas há pouca informação sobre estudos que aplicaram os potenciais evocados auditivos corticais nessa população. Isso ressalta a necessidade de investigações adicionais para aprofundar o conhecimento sobre os efeitos do CMVc no processamento auditivo central. É fundamental investir em pesquisas futuras que explorem o impacto do CMVc na maturação e no funcionamento das vias auditivas em nível cortical, contribuindo para uma compreensão mais abrangente e para o desenvolvimento de estratégias diagnósticas e terapêuticas mais eficazes.

## CONCLUSÃO

Crianças com CMVc assintomático podem estar em risco para alterações no processamento auditivo central. Nosso estudo evidenciou diferenças na habilidade dessas crianças, caracterizadas por morfologia indefinida, menores latências de N1 e P2, e maior amplitude de N2, quando comparadas a crianças da mesma idade sem exposição ao vírus.

O estudo reforça a importância do PEAC como ferramenta para avaliar essas alterações e destaca a necessidade de pesquisas futuras para compreender melhor os efeitos do CMVc no desenvolvimento auditivo central.
